# The prevalence, virulence, and serogroups of *Dichelobacter nodosus* and prevalence of *Fusobacterium necrophorum* in footrot lesions of sheep and cattle in Morocco

**DOI:** 10.14202/vetworld.2023.668-674

**Published:** 2023-04-03

**Authors:** Zahra Bamouh, Z. Elkarhat, Z. Zouagui, O. Fassi Fihri, M. Elharrak

**Affiliations:** 1 Department of Research and Development, Multi Chemical Industry Santé Animale, Lot. 157, Z.I., Sud-Ouest (ERAC) B.P: 278, Mohammedia, 28810, Morocco; 2 Department of Microbiology, Immunology and Contagious Diseases Institute of Agronomy and Veterinary Hassan II, Rabat, Morocco

**Keywords:** cattle, *Dichelobacter nodosus*, footrot, *Fusobacterium necrophorum*, polymerase chain reaction, sheep

## Abstract

**Background and Aim:**

Footrot is a contagious disease of ruminants leading to severe economic losses. This study aimed to estimate the prevalence, virulence, and serogroups of *Dichelobacter nodosus* and the prevalence of *Fusobacterium necrophorum* in footrot lesions of sheep and cattle.

**Materials and Methods:**

A total of 106 pathogenic lesion samples were taken from 74 sheep and 32 cattle exhibiting typical footrot lesions and were analyzed for the presence of *D. nodosus* and *F. necrophorum* by real-time polymerase chain reaction (PCR). Both virulence and serogroup were estimated for *D. nodosus* positive samples.

**Results:**

Among the 106 samples, 89 were positive by PCR for *F. necrophorum*, *D. nodosus*, or both. *Dichelobacter nodosus* was detected at a rate of 78.3% versus 28.3% for *F. necrophorum*. Virulent *D. nodosus* strains were detected in 67.5% of positive samples, with a higher rate in sheep (73.4%) than in cattle (47.4%). Benign *D. nodosus* strains were detected in 57.8% of samples, with a lower prevalence rate in sheep (50%) than in cattle (84.2%). The positive samples of *D. nodosus* revealed the presence of three dominant serogroups (D, H, I) and three minor serogroups (G, C, A) by serogroup-specific multiplex PCR.

**Conclusion:**

The findings provided information on the prevalence of *D. nodosus* and *F. necrophorum* strains in footrot lesions of sheep and cattle in some regions of Morocco, which will be useful for developing an effective autovaccine for the prevention of this disease in cattle and sheep in these regions.

## Introduction

Footrot is a highly contagious disease of ruminants caused by several bacterial species, including *Dichelobacter nodosus* as the main etiological agent and *Fusobacterium necrophorum* as the second pathogen to induce synergically footrot in animals [1–3]. The disease is favored by environmental factors, such as warm and wet weather, pasture quality, nutrition, and animal density [4–6]. The disease causes lameness and significant production and economic losses worldwide, in addition to a considerable impact on animal welfare [7].

*Dichelobacter nodosus* is divided into virulent and benign strains that are associated with different forms of the disease and the bacteria are classified into ten serogroups (A–I and M) based on fimbrial antigen, encoded by the 45 fimA gene and their distribution varies from place to place [8, 9]. Detection of *D. nodosus* is carried out by polymerase chain reaction (PCR) using specific primers that can differentiate benign from virulent strains, while serogrouping is carried out by multiplex PCR using *fimA* gene-specific primers [10, 11].

*Fusobacterium necrophorum* is associated with different diseases in animals and humans and pathogenicity is based on several virulence factors such as leukotoxin, hemolysin, and hemagglutinin that play an essential role in the infection process [12].

Footrot has been notified as the main cause of lameness in ruminants. The prevalence of footrot lesions has been reported in many countries, such as United Kingdom (8–10%) [13], Bhutan (3.1%) [14], India (12–15%) [15–17], Sweden (5.8% in slaughter lambs with score ≥2) [18], Germany (42.93%) [19], Bangladesh (4.4%) [20], The prevalence of lameness in general in dairy cows has been investigated clinically in Algeria (13%) [21].

Footrot is a multifactorial disease and treatment used for control, such as foot bathing and the use of antibiotics, is costly and provides only temporary remission by repeated treatment [22–25]. Furthermore, the large use of antibiotics in such diseases raises the issue of antibiotic resistance [26, 27]. The disease can be prevented by the application of vaccines but it is complicated due to the presence of several serogroups of *D. nodosus* in the same flock, with no cross-immunity between serogroups, leading to unsatisfactory results [25, 28]. Footvax^®^ (MSD Animal Health, UK) is the only licensed multivalent vaccine against footrot in the UK based on nine fimbrial serogroups of *D. nodosus* that produces short-term antibody responses due to antigenic competition that reduces vaccination efficacy [24, 29].

At present, research is focused on developing autovaccines or serogroup-specific vaccines for use during specific footrot outbreaks [30–33]. This approach needs the identification of circulating bacterial strains and serogrouping of *D. nodosus* circulating in the flock, followed by developing a serogroup-specific vaccine [16, 34]. The prevalence of footrot and its causative agent has been the subject of different studies in several parts of the world. In Morocco, the lameness due to footrot lesions has not been sufficiently researched. To our knowledge, studies to estimate the serogroups of *D. nodosu*s have not been conducted yet. The only recent study by Sidki [35] described the epidemiological and molecular results of digital infections, specially footrot in sheep.

Therefore, this study aimed to estimate the prevalence of *F. necrophorum* and *D. nodosus* strains in footrot lesions of sheep and cattle with the identification of dominant serogroups of *D. nodosus*.

## Materials and Methods

### Ethical approval

This study did not require official or institutional ethical approval. The animals were handled according to high ethical standards.

### Study period and location

The study was conducted during mild and humid weather in the winter and spring of 2021 (November and May) in some regions of Morocco based on epidemiological inquiries to evaluate the presence of footrot in the region. This sampling period was chosen with regard to favorable environmental conditions for disease expression and transmission.

### Inspection of animals

**Figure-1:**
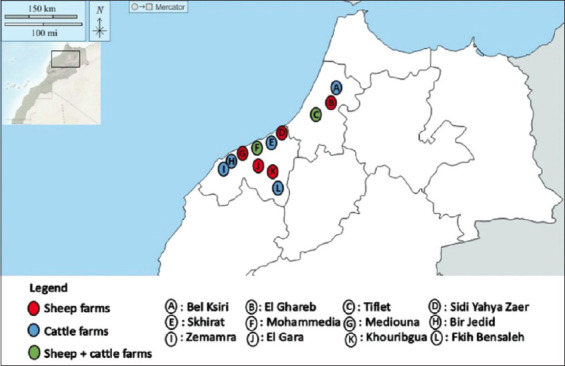
Geographical distribution of sites where footrot lesion samples were collected from sheep and cattle in Western Morocco [Source: d-maps.com].

The selection criteria of farms included the presence of clinical cases of footrot, flocks with no <30 animals, and collaboration of the farmer. A total of 22 sheep flocks with 6430 sheep and eight cattle flocks with a population size of 2480 in mid-West Morocco were inspected for footrot lesions (Figure-1). This is a selection of representative farms with a history of footrot and the presence of cases at the time of visit. In all flocks investigated, cases of lameness were observed and recorded by farm staff and footrot was confirmed by the veterinary practitioner supervising the farms concerned.

### Sample collection

All four animal feet were examined visually for footrot lesions and a score was assigned to each foot of each animal between 1 and 5 as described by Stewart and Claxton [36] for sheep, and by Döpfmer and Guggenmoos-Holzmann [37] for Cattle. A total of 106 samples of pathogenic lesions were collected on 74 sheep and 32 cattle suspected of footrot based on clinical signs and with lesions scores ranging from 2 to 4. Veterinarian performed the sample collection. Sheep were placed in lateral recumbency and cattle in foot trimming crush. Foot lesion samples were collected from the interdigital skin between the horn of the hoof and the sensitive underlying tissue using a sterile cotton-tipped swab. Only one affected foot was sampled from each affected animal for PCR analysis.

### Data collection

For each collected footrot lesion sample, the following information was recorded, including animal identification (species, sex, and age), date of sampling, location of the farm, number of feet affected, and lesion score. In addition, a questionnaire was completed by the farm owner regarding herd size, access to pasture, history, and treatment of footrot.

### DNA extraction

DNA was extracted from collected swab samples using a DNA extraction kit (isolate II genomic DNA kit, Bioline, UK). 2 mL of sterile phosphate-buffered saline (PBS) was added to swabs and vortexed for 1 min. A 200 μL mixture of each swab-PBS sample was used for DNA extraction according to the manufacturer’s recommendations.

The negative and positive controls used for PCR were kindly provided by the ruminant’s unit of the Hassan II Agronomic and Veterinary Institute of Rabat, Morocco.

### Polymerase chain reaction for the detection of *F. necrophorum*

All samples were tested for *F. necrophorum*, as described previously by Jensen *et al*. [38]. Briefly, the real-time PCR assay (quantitative PCR [qPCR]) was performed using 20 μL reaction mixtures containing 10 μL of Luna Universal probe qPCR Master Mix, 2yl of 300 or 400 nM (final concentration) of the forward and reverse primer, 1 μL of 300 or 400 nM (final concentration) of the probe, and 5 μL of template DNA. Thermal cycling on a Quant Studio 1 system using the following amplification program, 10 min at 95°C, followed by 45 cycles of 15 s at 95°C and 60 s at 60°C.

### Dichelobacter nodosus detection

All swab samples were analyzed for *D. nodosus* by a qPCR assay targeting the *aprV2* and *aprB2* genes as described previously by Frosth *et al*. [2], with some modifications. The qPCR assay was carried out in 20 μL reaction mixtures containing 10 μL of Luna Universal probe QPCR Master Mix (Biolabs, UK), 400 nM of each primer, 100 nM of each probe, and 4 μL of template DNA. The performance of the qPCR assay involved an initial denaturation step at 95°C for 5 min, followed by 45 cycles of 95°C for 15 s and 60°C for 60 s.

### Serogrouping of *D. nodosus*

*Dichelobacter nodosus* serogrouping was performed using the multiplex PCR of nine (A–I) serogroup-specific primers as described previously by Dhungyel *et al*. [10]. The PCR products were analyzed in 2% agarose gels and visualized under ultraviolet illumination using an E-Box Vilber (Vilber, France).

### Statistical analysis

Data analysis was performed using the Chi-square test. Results of the prevalence of *D. nodosus* and *F. necrophorum* in sheep were compared with the results in cattle. In addition, a comparison between the prevalence of virulent and benign *D. nodosus* in cattle and sheep was carried out. Values of p < 0.05 were considered significant.

## Results

### Clinical observation

In total, 106 interdigital swab samples were collected from 30 farms. The feet examination showed healthy interdigital skin (Score 0) in 6356 sheep and 2448 cattle. Interdigital dermatitis (Scores 1 and 2) was found in 34 sheep and 14 cattle. There were 40 sheep and 18 cattle that showed a separation of hoof horn from the underlying dermis (Scores 3–4) (Table-1). Herd prevalence ranged between 0.7%–13.3% in sheep and 0.7%–7.5% in cattle.

**Table-1:** Summary of the severity of footrot lesions based on a scoring system and the number of animals that were positive for *D. nodosus* and *F. necrophorum* or both bacterial species.

Species	Sampled sick animals	Footrot severity					*F. necrophorum* +or/and *D. nodosus+*	*Only D. nodosus+*	*Only F. necrophorum+*	*F. necrophorum*+ and *D. nodosus+*

		Score 1	Score 2	Score 3	Score 4	Average score				
Sheep	74	0	34	35	5	2.6	65	52	1	12
Cattle	32	0	14	14	4	2.7	24	7	5	12
Total	106	0	48	49	9	2.65	89	59	6	24

*D. nodosus=Dichelobacter nodosus, F. necrophorum=Fusobacterium necrophorum*

Clinical observations showed that adult cattle (100%) and sheep (92%) were more affected by lameness due to footrot lesions. All visited cattle farms had dairy cows with no access to pasture, whereas among 22 sheep flocks visited, ten had access to pastures. Most animals sampled were affected only in one foot (88% in sheep and 94% in cattle). Animals with a score of one were not found in this study (Table-1). Recorded scores range from 2 to 4 in sheep and cattle and the highest percentage was noted in scores 2 and 3 regardless of species. Only a few animals were observed with a score of 4, or the most severe lesions (Table-1).

### Molecular analysis

Among the 106 samples obtained from the 74 sheep and 32 cattle and analyzed by qPCR, 89 were positive for either *F. necrophorum*, *D. nodosus* or both (84%), and 17 were negative (16%). The percentage of coinfections with these two bacteria was 16.2% in sheep and 37.5% in cattle. Out of the 89 positives samples, 66.3% (59/89) were positive for *D. nodosus*, 6.7% (6/89) for *F. necrophorum*, and 27% (24/89) for both *F. necrophorum* and *D. nodosus* (Table-1). Out of the 83 samples positive for *D. nodosus*, virulent strains were detected in 56 samples (67.5%), benign strains in 46 samples (55.4%), and both virulent and benign strains were present in 19 samples (22.9%) (Table-1).

Among sheep, 64 of 65 samples were positive for *D. nodosus*, while only 13 of 65 samples were positive for *F. necrophorum*. In cattle, 19 of 24 samples were positive for *D. nodosus* and 17 of 24 were positive for *F. necrophorum* (Table-1). A significant difference was observed regarding the prevalence of *D. nodosus* (p = 0.002) and *F. necrophorum* (p = 0.0002) between sheep and cattle.

The prevalence of virulent *D. nodosus* (*aprV2+*) in sheep was higher than in cattle, 53.1% (34/64) versus 15.8% (3/19), respectively. In contrast, the prevalence of benign infection associated with *D. nodosus* (*aprB2+*) was lower in sheep [26.6% (17/64)] than in cattle [52.6% (10/19)] (Table-2). Virulent *D. nodosus* was statistically higher (p = 0.03) in sheep compared to cattle; however, benign *D. nodosus* was significantly higher (p = 0.008) in cattle than sheep.

**Table-2:** The number of sheep and cattle positive for virulent (*aprV2*+) and benign (*aprB2*+) *D. nodosus*.

Species	No. of samples	Positive to *D. nodosus*	Prevalence	*AprV2*+	Prevalence	*AprB2*+	Prevalence	Mixt *AprV2*+ *AprB2*+	Prevalence
Sheep	74	64	86.5% (p *=* 0.02)	34	53.1% (p = 0.03)	17	26.6%	13	20.3%
Cattle	32	19	59.3%	3	15.8%	10	52.6% (p = 0.008)	6	31.6%
Total	106	83	78.3%	37	44.6%	27	32.5%	19	22.9%

*D. nodosus=Dichelobacter nodosus*

In sheep, benign *D. nodosus* was detected in 17 animals with score 3, and virulent *D. nodosus* was detected in only three animals with score 4. However, in cattle, animals with score 4 were negative for *D. nodosus* by PCR. The three animals positive to *ApV2+* have a score of 2 and six animals positive to *AprB2+* have score of 3.

From the 83 positive samples for *D. nodosus*, serogroups were identified in 31 samples and showed the dominance of 3 serogroups (D, H, and I), with D dominant in sheep and H in cattle (Table-3). In sheep, serogroups of *D. nodosus* were identified in virulent strains. Serogroups C and A were noted only in cattle. Among 31 samples, 21 were positive for a single serogroup, while ten samples were positive for more than one serogroup.

**Table-3:** Distribution of *D. nodosus* serogroups in sheep and cattle.

Species	Number	Serogroups										

		D	H	I	G	C	A	D+H	H+I	D+G	C+H	A+C+D
Sheep	16	9	2	6	1	0	0	0	1	1	0	0
Cattle	15	6	9	5	1	2	1	3	2	1	1	1
Total	31	15	11	11	2	2	1	3	3	2	1	1

*D. nodosus=Dichelobacter nodosus*

In sheep, serogroups of *D. nodosus* were detected in three sampled areas, first area C (66.7%), followed by area D (22.2%) and in the last area K (11.1%). In cattle, the distribution of serogroups of *D. nodosus* serogroups was specific to three sampled areas A (62.5%), C (21%), and I (16.7%). Zone “C” is common between sheep and cattle.

## Discussion

This study aimed to estimate the prevalence of strains of *D. nodosus* and *F. necrophorum* in footrot lesions in sheep and cattle, identification of dominant serogroups of *D. nodosus*, and related virulence. However, several limitations exist in this study that should be addressed with future research. First, samples were not taken from healthy feet to compare bacteria found in diseased feet with those from healthy. Given that the strains are also present in not affected feet [26]. Second, our data come from small herds; hence, this study population cannot be representative of Moroccan herds in general.

This study showed that *D. nodosus* was present in collected samples at a rate of 78.3% versus 28.3% for *F. necrophorum* and the percentage of infection with only *F. necrophorum* was the lowest at 6.7%. In this study, the percentage of coinfections with two bacteria strains was higher in cattle than sheep.

All collected samples were taken from animals with lameness and footrot lesions; however, 16% of samples were negative for both bacteria species. An explanation for the negative samples was that the animals were either in a healing phase or the infection was due to another cause. In our study that investigated pasture flocks of sheep, we found *D. nodosus* strain to be dominant, while in intensive cattle farms, *F. necrophorum and D. nodosus* were equally prevalent, thus suggesting that the husbandry system impacted the prevalence of the bacterial pathogens.

Virulent *D. nodosus* strains were detected in 67.5% of positive samples, being higher in sheep (73.4%) than in cattle (47.4%). This study shows the coexistence of benign and virulent strains in the same samples and when analyzing the prevalence of virulent and benign *D. nodosus* strains in sheep, 73.4% of samples were associated with a virulent strain, while 50% of samples were associated with benign strain, thus demonstrating that the two bacteria species coexisted in the same lesions. However, 17 lesions were associated with the benign strain only. There is no correlation between the severity of lesions and prevalence of the clinical scores. However, in 24 positive cattle samples, infection was associated more frequently with *D. nodosus* and *F. necrophorum* in 50%, 29.2% with *D. nodosus* alone, and 21% with *F. necrophorum* alone. However, in the study of Albuquerque *et al*. [39], the coinfection with both bacteria was associated with greater disease severity.

The results of this study detected six serogroups of *D. nodosus* (D, H, I, G, C, and A) with the dominance of D (48.3%), and H and I serogroups (35.5% each). Serogroups seem to be linked to specific geographical localization, but more samples and other regions need to be investigated to confirm this observation.

Several studies in Bhutan [14], India [15], Portugal [39], Australia [40], Great Britain [41], and New Zealand [42] reported dominance of serogroup B of *D. nodosus* which was not present in this study. In addition, ten samples among 31 were positives for two or three serogroups of *D. nodosus* simultaneously, which was in agreement with other studies that reported mixed infection with several serogroups on a single footrot lesion [33, 39]. These findings provide valuable information for developing herd-specific vaccines, which are made from isolated bacteria in the region and are more efficient than multivalent commercial vaccines [33, 42].

Footrot is a serious constraint in ruminants’ breeding and control by vaccination needs an in-depth understanding of the etiological bacterial pathogens circulating in each region. This study provides detailed information on the prevalence of *D. nodosus* and *F. necrophorum* associated with causes of footrot among sheep and cattle in Morocco. The *D. nodosus* virulent strain was dominant in sheep and had an equal prevalence of both species, *D. nodosus* and *F. necrophorum* strains, in cattle.

## Conclusion

Overall, the findings of this study provide the first understanding of the bacterial causes of footrot in sheep and cattle in Morocco, which is critical for developing an effective vaccine to prevent this globally important disease.

## Authors’ Contributions

ZB: Performed the experiments, analyzed the data, and drafted the manuscript. ZE: Carried out analysis and interpretation of data and participated in the writing of the manuscript. OFF and ZZ: Conceptualization and design of the study and participated in the writing of the manuscript. ME: Designed the study, data analysis and interpretation, and drafted the manuscript. All authors have read, reviewed, and approved the final manuscript.
